# YouTube as a digital health platform: A cross-sectional thematic and sentiment analysis of the 100 most-viewed knee osteoarthritis videos

**DOI:** 10.1097/MD.0000000000047925

**Published:** 2026-02-28

**Authors:** Nurmuhammet Taş, Yakup Erden, Mustafa Hüseyin Temel, Fatih Bağcier

**Affiliations:** a Clinic of Physical Medicine and Rehabilitation, Erzurum City Hospital, Erzurum, Turkey; b Clinic of Physical Medicine and Rehabilitation, İzzet Baysal Physical Medicine and Rehabilitation Training and Research Hospital, Bolu, Turkey; c Clinic of Physical Medicine and Rehabilitation, Univeristy of Health Sciences, Sultan 2 Abdulhamid Han Training and Research Hospital, İstanbul, Turkey; d Clinic of Physical Medicine and Rehabilitation, Private Clinic, İstanbul, Turkey.

**Keywords:** digital health platforms, knee osteoarthritis videos, sentiment analysis YouTube, social media in healthcare, YouTube health information

## Abstract

Knee osteoarthritis (OA) is a prevalent source of pain and disability. As YouTube becomes increasingly used for health information, the nature and impact of OA-related content warrant systematic evaluation. Using the YouTube Data Application Programming Interface, 327 videos were initially retrieved with the terms “knee osteoarthritis,” “gonarthrosis,” and “degenerative joint disease.” After applying exclusion criteria, the 100 most-viewed English-language videos were selected. Metadata and 60,888 user comments were extracted. Videos were thematically categorized into 6 domains: treatment, disease education, symptoms, diagnosis, patient experience, and uncategorized. Sentiment analysis was performed using the TextBlob library, and statistical trends were assessed via Statistical Package for the Social Sciences (SPSS) 29.0. Treatment was the dominant theme (38%), with nonsurgical options like exercise and rehabilitation comprising over half of these videos (55.3%). Educational (24%) and symptom-related content (12%) were also frequent. Viewer engagement peaked in 2022, with the highest number of views and comments. Over time, video uploads and comment activity increased, best modeled by quadratic trends. Seasonal variation in engagement was not significant. Commonly used words included “exercise,” “knee,” “thank,” and “pain.” Sentiment analysis revealed a decline in positive comments and a rise in neutral sentiment, while negative sentiment remained stable. YouTube serves as a growing platform for knee OA information, emphasizing conservative treatments. Although engagement and content volume have increased, sentiment has shifted toward neutrality. Given the variability in content quality, healthcare providers should guide patients toward credible, evidence-based resources to optimize digital health literacy.

## 1. Introduction

Knee osteoarthritis (KOA), a leading cause of chronic pain and functional limitation in adults, particularly in aging populations, is a degenerative joint disorder marked by progressive cartilage loss, subchondral bone remodeling, synovial inflammation, and impaired biomechanics.^[1,2]^ As a primary contributor to global disability and reduced mobility, KOA not only compromises physical independence but also exerts substantial psychosocial and economic burdens on individuals and healthcare systems alike.^[3–5]^ In the absence of definitive curative treatments, patients often navigate a complex landscape of symptom management strategies, seeking both medical interventions and self-care approaches to mitigate pain and preserve function.^[6–8]^

Amid this clinical reality, the digital era has dramatically transformed the way patients engage with health information. YouTube, in particular, has emerged as a dominant multimedia platform where individuals can access and contribute to health-related discourse. Its global accessibility, visual format, and algorithm-driven recommendations make it a powerful medium for sharing medical explanations, treatment testimonials, exercise demonstrations, and patient experiences. However, the largely unregulated, user-generated nature of YouTube content introduces considerable variability in scientific accuracy, clinical relevance, and reliability (posing challenges to health literacy and informed decision-making).^[9–13]^

While the academic community has increasingly turned its attention to online health narratives in areas such as oncology, dermatology, and rare diseases, chronic musculoskeletal conditions like KOA remain underrepresented in digital discourse analyses. Yet, the magnitude of KOA’s impact and the breadth of public engagement it stimulates suggest a pressing need to evaluate how this condition is represented and discussed online. YouTube comments, in particular, offer a unique window into the lived experiences, emotional responses, expectations, frustrations, and knowledge gaps of patients and caregivers. These narratives are not merely peripheral: they often reveal critical insights into how individuals interpret symptoms, perceive treatment effectiveness, and assess the quality of care options.^[14–18]^

This study seeks to systematically explore the evolving digital conversation surrounding KOA on YouTube. By focusing on the top 100 most-viewed publicly available videos, we conducted a longitudinal analysis of user engagement (measured via comment volume, linguistic content, sentiment orientation, and thematic focus) to map the trajectory of public discourse over time. By identifying recurring patterns, unmet informational needs, and sentiment shifts, this research contributes to the growing field of digital epidemiology and health communication. Ultimately, our findings aim to inform clinicians, educators, and policymakers about the opportunities and challenges presented by digital platforms in shaping patient perspectives and guiding health-seeking behavior in the context of chronic joint disease.^[1,14,15,19]^

## 2. Materials and methods

### 2.1. Video selection and inclusion criteria

Videos were initially retrieved from YouTube using the search terms “osteoarthritis,” “gonarthrosis,” and “degenerative joint disease” through the YouTube Data Application Programming Interface (API).^[20]^ The resulting list was sorted by view count in descending order, and the top-ranking videos were sequentially reviewed by a research team (NT and FB). Inclusion was not limited to metadata-matching results; instead, a 2-tiered strategy was employed. First, videos were retained if the relevant keywords appeared in the title, description, or tags. Second, videos that lacked explicit keyword matches but whose visual or verbal content demonstrated clear thematic relevance to degenerative joint disease were also included. This interpretative layer enabled the inclusion of videos discussing OA under broader or alternate terminologies. In cases of disagreement during content evaluation, a consensus was reached under the supervision of a 3rd reviewer (MHT), ensuring consistency and thematic integrity.

A total of 327 publicly accessible videos were obtained during the initial extraction. The inclusion criteria for video eligibility were:

- Videos must be publicly accessible and not age-restricted.
- Videos must contain at least 1 publicly visible user comment.
- Duplicate, private, or deleted videos were excluded from the dataset.

**Figure 1.**
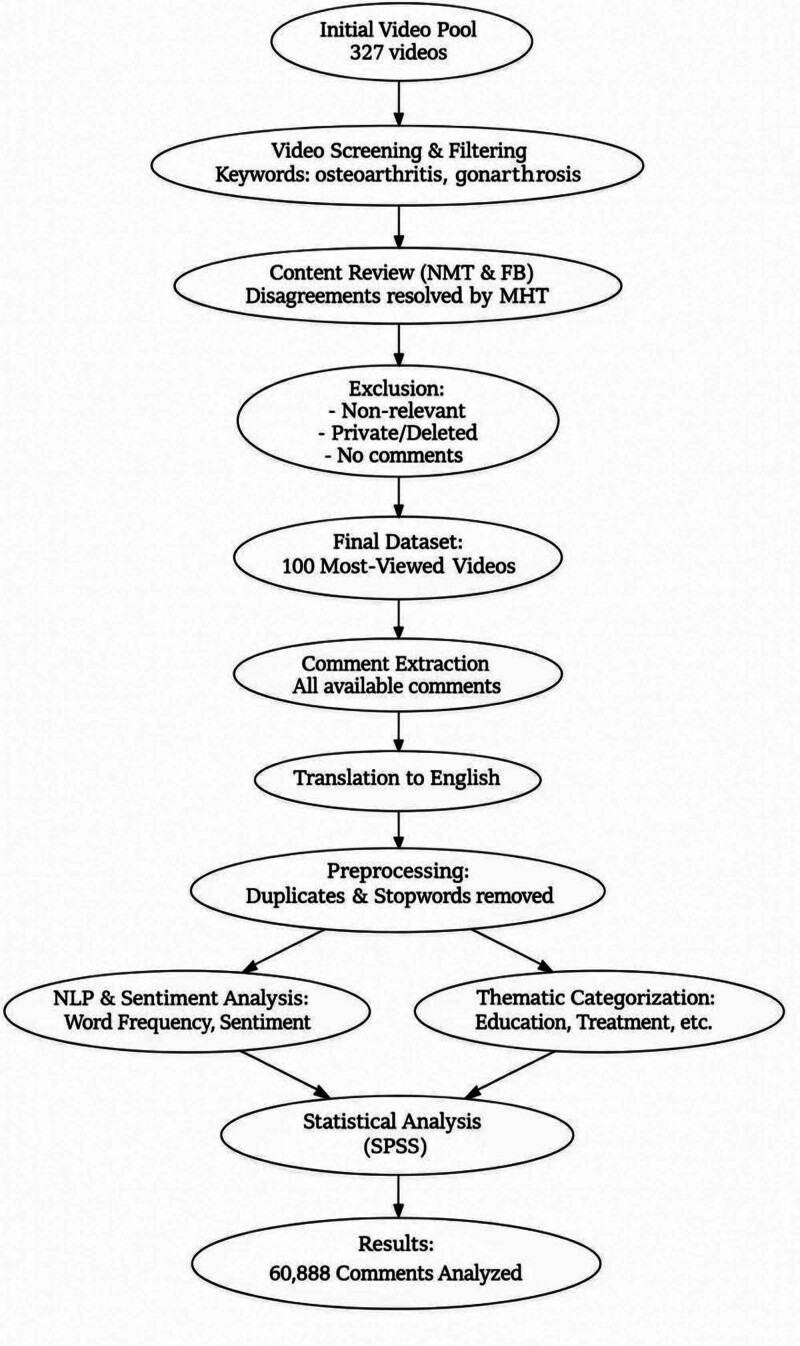
Diagram of high-impact content with significant user engagement reflecting the popular discourse on knee osteoarthritis on YouTube.

After applying these criteria, the top 100 most-viewed videos were selected for further analysis. This strategy ensured the analysis focused on high-impact content with significant user engagement, reflective of popular discourse on knee OA on the YouTube platform (Fig. 1).

### 2.2. Comment extraction and preprocessing

For each of the top 100 videos, all available user comments were extracted using the YouTube Data API. Extracted metadata included:

- Comment text.
- Comment ID.
- Associated video ID.

A total of 60.888 user comments were collected. To prepare the text corpus for downstream analysis, a multistage preprocessing pipeline was applied:

- Duplicate removal: identical comments were filtered using strict string-matching logic.
- Language filtering: all comments, regardless of original language, were retained and subsequently translated into English using automated translation tools. This approach enabled inclusive analysis of user discourse regardless of native language.
- Stopword removal: generic stopwords (e.g., “the,” “is,” “at”) were removed using the Natural Language Toolkit stopword list to enhance semantic clarity.
- Exclusion of low-value comments: non-informative comments (those consisting solely of emojis, punctuation, or very short expressions (e.g., “ok,” “👍”)) were excluded.
- Spelling normalization: minor spelling corrections and lowercase conversion were applied to improve linguistic uniformity.

The final dataset consisted of cleaned, unique, semantically meaningful, and uniformly translated English-language comments. This refined corpus provided a robust foundation for both quantitative and qualitative textual analysis.

### 2.3. Natural Language Processing pipeline

The cleaned comment dataset was subjected to a structured Natural Language Processing workflow for linguistic and sentiment analysis.^[21]^

- Normalization: all text was converted to lowercase, and punctuation and non-alphanumeric characters were removed using regular expressions.
- Stopword and custom word removal: standard English stopwords were excluded using the Natural Language Toolkit library. However, for word frequency visualization (word cloud), only standard stopwords were removed, preserving domain-relevant and frequently used terms in user discourse.^[22]^
- Tokenization: comments were split into individual words (unigrams) to prepare for downstream analyses such as frequency calculation, topic detection, and sentiment scoring.

### 2.4. Thematic categorization of video content

To assess the thematic diversity of the analyzed videos, 2 independent reviewers (NT and FB) manually examined the content and classified each video into 1 of 6 primary thematic categories:

- Disease education.
- Symptoms.
- Diagnosis.
- Treatment.
- Patient experience.
- Miscellaneous.

Subsequently, all videos categorized under the “Treatment” theme were reexamined and further subclassified into 3 primary treatment modalities:

- Surgical.
- Exercise and rehabilitation.
- Alternative methods.

In cases where the video content did not align clearly with any of these categories, it was labeled as uncategorized. Discrepancies in classification were resolved by consensus or, when necessary, by a 3rd reviewer (MHT). This layered thematic framework allowed for more detailed insights into the treatment discourse.

### 2.5. Sentiment analysis

To examine the emotional tone embedded in user comments, a sentiment analysis was performed on the cleaned English-language corpus. Prior to analysis, all comments (regardless of their original language) were translated into English using automated translation tools to ensure linguistic consistency across the dataset.

Polarity scores were computed using the TextBlob library, which internally relies on a lexicon-based sentiment analysis algorithm derived from the Pattern library. Each word in a comment is compared against a predefined sentiment lexicon that assigns polarity values between –1.0 (strongly negative) and +1.0 (strongly positive). These values are aggregated across the sentence or comment while taking into account syntactic structures such as negations (e.g., “not good” reduces positivity) and modifiers (e.g., “very painful” increases negativity). The final polarity score is calculated as the normalized average of these word-level sentiment values. Based on this aggregate polarity score, comments were labeled as positive (score > 0.1), negative (score < –0.1), or neutral (–0.1 to 0.1).^[23]^

### 2.6. Quantitative and seasonal analysis

Video-level metadata including total view counts, video durations, total video counts, total comment counts, word counts mean number of comments per video, and mean number of words per comment were quantitatively analyzed. In addition, a seasonal comparison was performed to evaluate temporal trends in user engagement. Based on the upload dates, videos were grouped into 4 seasons (Winter, Spring, Summer, and Fall).

In order to quantify and visualize potential trends, linear and quadratic regression models were fitted to the yearly data for each metric. Goodness-of-fit values (*R*^2^) were calculated to assess the explanatory power of the models. These regression curves provided an interpretable view of whether engagement and keyword usage increased, declined, or followed nonlinear patterns over time.

Descriptive statistics were computed for each parameter, including:

- Mean, standard deviation, minimum, and maximum values.

### 2.7. Statistics

All statistical analyses were performed using IBM Statistical Package for the Social Sciences (SPSS Statistics, Version 29.0) (IBM Corp., Armonk). Descriptive statistics (mean, standard deviation, minimum, and maximum values) were calculated for continuous variables such as the number of comments per video and the number of words per comment. The normality of data distribution was assessed using the Shapiro–Wilk test. Depending on the distribution characteristics, parametric tests (1-way analysis of variance) or nonparametric equivalents (Kruskal–Wallis *H* test) were applied to compare seasonal differences. Additionally, a chi-square (χ^2^) test was employed to examine potential associations between categorical variables such as sentiment categories and seasons. A significance level of *P* < .05 was adopted for all inferential tests.

### 2.8. Ethical considerations

This study was conducted using only publicly available data from YouTube. No attempt was made to collect personally identifiable information, and all data were handled in accordance with ethical guidelines for digital and social media research. As all analyzed data were publicly accessible and no personally identifiable information was collected, institutional review board approval was not required in accordance with established guidelines for research using public-domain digital data.

## 3. Results

**Figure 2.**
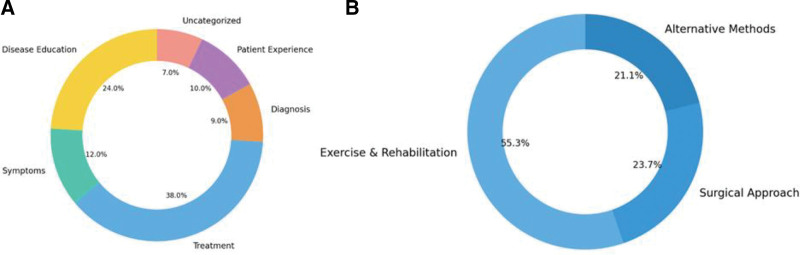
*C*ategorized version of videos.

A total of 327 videos were initially retrieved from YouTube using the search terms “osteoarthritis,” and “gonarthrosis” through the YouTube Data API. Of these, the 100 most watched videos were selected according to the inclusion criteria (Fig. 1.). YouTube videos were identified between 2011 and 2025, from which 60.888 user comments were collected. Thematic analysis showed that most videos focused on treatment (38%), especially nonsurgical methods like exercise and rehabilitation (55.3% of treatment videos). Disease education (24%) and symptoms (12%) were also common, while patient experiences, diagnosis, and uncategorized topics were less frequent (Fig. 2). The year with the most videos watched was 2022 (n = 40.992.742). The highest number of comments was also recorded in 2022 (n = 16.125) (Table 1).

**Table 1:** Annual distribution of the top 100 most-viewed knee osteoarthritis videos on YouTube.

Number	Years	View count	Comment count	Number of videos by year
1	2011	1.387.794	141	2
2	2012	860.558	355	1
3	2013	1.529.422	24	1
4	2014	269.647	0	1
5	2015	0	0	0
6	2016	5.929.599	2.771	7
7	2017	7.170.524	929	2
8	2018	1.113.850	335	2
9	2019	8.306.680	4.796	3
10	2020	8.655.738	6.789	2
11	2021	9.555.078	3.595	4
12	2022	40.992.742	16.125	26
13	2023	5.369.671	12.872	22
14	2024	39.378.300	11.876	23
15	2025	2.586.963	280	4

**Figure 3.**
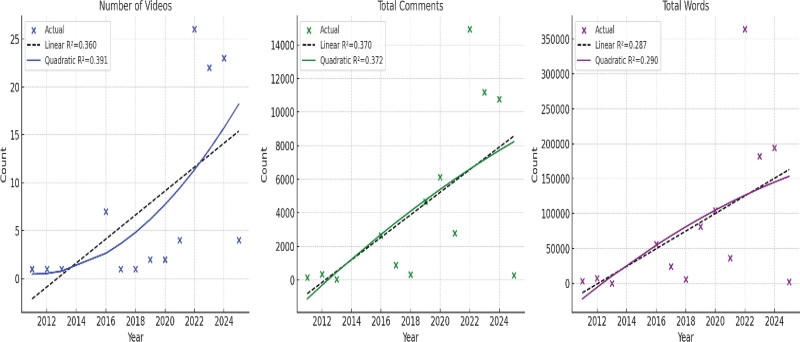
Variation in total word count of comments, comment length, and interaction depth over the years.

The temporal distribution of OA-related YouTube content revealed an overall increasing trend over the years (Fig. 2). The number of videos showed a modest growth, with a better fit for the quadratic model (*R*^2^ = 0.391) compared to the linear model (*R*^2^ = 0.360), indicating a slight acceleration in video uploads in recent years. Similarly, the total comment counts exhibited a progressive increase, again favoring a quadratic trend (*R*^2^ = 0.372), suggesting gradually rising viewer engagement. The total word count of comments also followed an upward trajectory, although with lower model fits (*R*^2^ = 0.290 for quadratic, *R*^2^ = 0.287 for linear), reflecting variability in comment length and engagement depth across years (Fig. 3).

When the data were analyzed across seasons, no statistically significant differences were found in either the average number of comments per video or the average word count per comment (*P* > .05) (Table 2).

**Table 2:** Seasonal analysis of youtube comments.

Season	Mean comment count ± SD (min–max)	Mean word count ± SD (min–max)	*P* value
Winter	712.1 ± 1020.2 (1–4129)	16.63 ± 26.71 (1–829)	.751
Spring	362.1 ± 296.7 (1–1144)	17.49 ± 29.29 (1–816)	
Summer	722.2 ± 946.3 (1–4164)	17.05 ± 29.26 (1–676)	
Fall	571.0 ± 969.8 (1–5098)	24.95 ± 39.78 (1–1516)	

A focused lexical analysis was performed to evaluate the prevalence of key terms frequently appearing in user comments related to knee OA. The most prominently featured words were “exercise” (n = 905), “knee” (n = 851), “thank” (n = 798), “pain” (n = 750), and “video” (n = 704).

**Figure 4.**
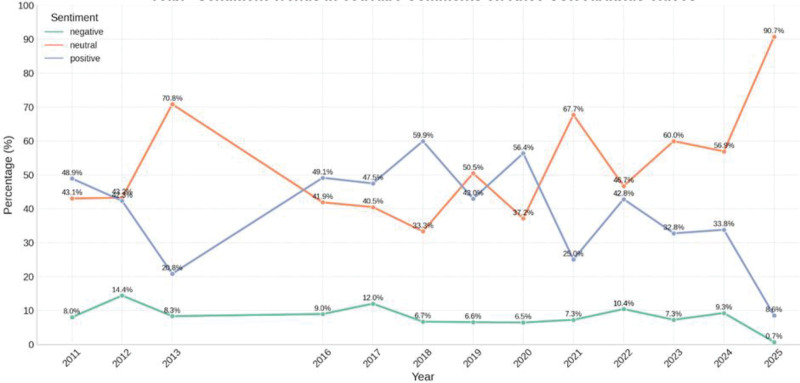
Temporal trends in sentiment distribution of YouTube comments related to knee osteoarthritis (2011–2025). The proportions of positive, neutral, and negative comments were calculated using sentiment polarity scores and demonstrate a gradual shift toward neutral sentiment over time.

Sentiment analysis revealed a decline in positive comments over time, from nearly 59.9 % in 2018 to under 8.6 % by 2025. Neutral sentiment fluctuated and reached its highest peak at 90.7% in 2025. Negative sentiment was more horizontal and was at its lowest level in 2025 (Fig. 4).

## 4. Discussion

With the increasing availability and use of digital platforms for health-related information, understanding public engagement with chronic conditions such as OA has become critically important. Osteoarthritis not only impairs quality of life in older populations but also poses a significant economic burden on healthcare systems worldwide.^[24]^ This study contributes to the growing body of digital health research by presenting a longitudinal analysis of user-generated YouTube comments after 2011, shedding light on the evolving discourse, sentiment, and language patterns associated with OA-related content.^[25]^ As social media increasingly serves as both a source of health information and a space for emotional and behavioral expression, platforms like YouTube offer a novel lens through which public engagement and health communication strategies can be examined.^[26,27]^ The consistent rise in comment volume and content length (particularly in recent years) highlights growing public interest in this condition and suggests potential gaps in formal patient education or clinical outreach efforts.^[28]^ This is the first study to systematically examine user-generated comments, offering a novel perspective on public discourse and emotional responses.

Although OA has long been recognized as a significant clinical and public health issue, relatively little is known about how this condition is represented in open-access digital communication channels. In recent years, YouTube has emerged not only as an educational tool but also as a mirror of public sentiment, offering unique insights into how individuals engage with complex medical issues.^[12,24]^ While comment-based analyses have been conducted in other health domains (such as polycystic ovary syndrome, pressure ulcers, cancer care, and COVID-19) no prior research has focused specifically on OA.^[29–31]^ Unlike traditional data forms, user comments on YouTube provide spontaneous, unfiltered responses that can reveal evolving patterns of interest, emotion, and understanding over time.^[32]^ Therefore, this study aims to analyze public discourse surrounding OA through the lens of YouTube engagement after 2011, and to uncover how collective awareness, emotional tone, and thematic focus have shifted within this online environment.

Over time, the increasing volume of user engagement suggests that digital content related to OA is not merely subject to short-term interest, but rather embedded within long-term processes of public awareness and information-seeking behavior. In our study, a clear upward trend was observed in both the number of YouTube videos and user comments related to OA between 2011 and 2025. The peak in video uploads in 2022, along with the highest recorded comment volume and word count that same year, indicates a deepening public engagement with the topic. The steady increase in the average number of words per comment over time further reflects a qualitative shift in engagement, as users provide more detailed narratives and reflections. This evolution in interaction may have been accelerated during the COVID-19 pandemic (a period marked by increased reliance on online health information and heightened public awareness of complications related to physical inactivity).^[33,34]^ Social media platforms saw dramatic rises in use during the pandemic, becoming key channels for health information dissemination: even as misinformation surged in parallel. Such a shift suggests that YouTube content, like academic publications, has the potential to gain renewed relevance and impact over time. Indeed, broader analyses confirm a significant increase in public interest in OA and related treatments over the last decade.^[35]^ Furthermore, YouTube has been recognized as a common (though often low‑quality) source of OA information: studies highlight both increased volume and variable reliability of videos on knee and shoulder OA.^[14,36]^ In response to this variability, recent research has started evaluating the role of artificial intelligence in medical imaging interpretation, including the use of large language models such as ChatGPT-4o for automated Kellgren–Lawrence grading of knee X-rays: an essential component in OA diagnosis and education.^[37]^ Our analysis highlights this temporal progression and underscores YouTube as an active, reflective space where health-related knowledge and emotion are collectively constructed and reshaped.

The present study found no statistically significant seasonal variation in user sentiment related to OA content on YouTube. A reasonable explanation is that OA, as a chronic and persistent health condition, is not expected to be influenced by episodic or seasonal fluctuations. However, the asynchronous and global nature of YouTube may dilute local seasonal effects, as content is consumed across regions with varying climates, healthcare infrastructures, and treatment practices. While seasonal dynamics are often observed in other medical topics (such as allergies, respiratory infections, or even mood disorders) the absence of such a pattern here highlights the enduring and universal relevance of OA education and advocacy in digital spaces.

This observation aligns with findings from large-scale analyses indicating that weather conditions can influence emotional expression on social media platforms. For instance, Baylis et al reported that various meteorological factors, including temperature and humidity, are associated with changes in sentiment expressed on Facebook and Twitter. However, such effects may be less pronounced on global platforms like YouTube, where content consumption spans diverse climatic regions. Furthermore, a systematic review and meta-analysis by Wang et al examined the relationship between weather conditions and OA pain. The study found that barometric pressure and relative humidity were positively correlated with OA pain intensity, while temperature was negatively correlated. These findings suggest that while weather can affect physical symptoms, the impact on emotional sentiment in online discussions may be less significant. In our study, the seasonal effect was not statistically significant to support this data.

Beyond seasonal consistency, the emotional trajectory over the past 14 years indicates a shift in sentiment tone. Initially dominated by positive comments, sentiment gradually transitioned toward neutrality, which became the prevailing tone starting in 2021. This may suggest a move toward more informative and reflective engagement, rather than emotionally charged reactions. A modest rise in negative sentiment during 2023 to 2024 may be attributed to emotionally intense content, such as clinical imagery or patient stories. These evolving patterns reflect a maturation of online discourse and underscore the need for content that is both accurate and emotionally sensitive. Notably, despite the potential for seasonal influence, sentiment remained remarkably stable across all 4 seasons. This stability highlights the persistent and noncyclical nature of public concern surrounding OA, positioning YouTube as a consistent and reliable platform for health-related engagement.

This study has several limitations that should be acknowledged. First, the analysis was limited to publicly available YouTube data, which may not fully capture personalized user interaction metrics such as watch time or exposure influenced by recommendation algorithms. Another limitation concerns the linguistic scope of the data: although English-language comments were predominant, the exclusion of non-English content may limit the generalizability of the findings to global audiences. Furthermore, the study did not account for the demographic or geographic background of commenters, which could offer critical insights into disparities in awareness, access to care, and health literacy. Lastly, although the study includes data post-2011, it did not analyze specific video metadata (such as creator type, video quality, or affiliation with health authorities) which could influence patterns of engagement and sentiment. Future research should aim to incorporate multilingual analysis, deeper metadata exploration, and cross-platform comparisons to gain a more comprehensive understanding of the digital discourse around OA.

## 6. Conclusion

This study offers a comprehensive examination of public discourse on OA over approximately 15 years, through the lens of YouTube content and user comments. The findings underscore the platform’s continued significance as both an educational resource and an emotional outlet for patients, caregivers, and healthcare professionals. Temporal and sentiment analyses revealed not only consistent engagement throughout the year, but also a gradual shift toward a neutral, informative discourse: highlighting the evolving maturity of online health communication. These insights emphasize the importance of designing content that balances clinical accuracy with emotional sensitivity. Future research should be expanded to include multilingual and cross-platform analyses, as well as deeper demographic and geographic profiling, to better understand the global dynamics of digital health engagement related to OA.

## Author contributions

**Conceptualization:** Fatih Bağcier.

**Data curation:** Nurmuhammet Tas.

**Formal analysis:** Nurmuhammet Tas.

**Investigation:** Mustafa Hüseyin Temel, Fatih Bağcier.

**Methodology:** Nurmuhammet Tas.

**Resources:** Fatih Bağcier.

**Software:** Nurmuhammet Tas, Fatih Bağcier.

**Validation:** Nurmuhammet Tas.

**Writing – original draft:** Nurmuhammet Tas.

**Writing – review & editing:** Yakup Erden, Fatih Bağcier.
